# Functionality, Implementation, Impact, and the Role of Health Literacy in Mobile Phone Apps for Gestational Diabetes: Scoping Review

**DOI:** 10.2196/diabetes.8045

**Published:** 2017-10-04

**Authors:** Qiong Chen, Elena T Carbone

**Affiliations:** 1 Department of Nutrition School of Public Health and Health Sciences University of Massachusetts/Amherst Amherst, MA United States

**Keywords:** gestational diabetes, mobile app, health literacy, smartphone, scoping review

## Abstract

**Background:**

The increasing ownership of mobile phones and advances in hardware and software position these devices as cost-effective personalized tools for health promotion and management among women with gestational diabetes mellitus (GDM). Numerous mobile phone apps are available online; however, to our knowledge, no review has documented how these apps are developed and evaluated in relation to GDM.

**Objective:**

The objective of our review was to answer the following 2 research questions: (1) What is known from the existing literature about the availability, functionality, and effectiveness of mobile phone apps on GDM prevention and management? (2) What is the role of health literacy in these apps?

**Methods:**

We searched 7 relevant electronic databases for original research documents using terms related to mobile phone apps, GDM, and health literacy. We thematically categorized selected articles using a framework adapted from Arksey and O’Malley.

**Results:**

We included 12 articles related to 7 apps or systems in the final analysis. We classified articles around 2 themes: (1) description of the development, feasibility, or usability of the apps or systems, and (2) trial protocols. The degree of personalization varied among the apps for GDM, and decision support systems can be used to generate time-efficient personalized feedback for both patients and health care providers. Health literacy was considered during the development or measured as an outcome by some apps.

**Conclusions:**

There is a limited body of research on mobile phone apps in relation to GDM prevention and management. Mobile phone apps can provide time- and cost-efficient personalized interventions for GDM. Several randomized controlled trials have been launched recently to evaluate the effectiveness of the apps. Consideration of health literacy should be improved when developing features of the apps.

## Introduction

Gestational diabetes mellitus (GDM) is diagnosed in the second or third trimester of pregnancy and is differentiated from type 1 (T1DM) and type 2 diabetes mellitus (T2DM) [1]. GDM affects 5.8% to 12.9% of women worldwide [2] and 9.2% of women in the United States on average [3]. Women with GDM are more likely than nondiabetic women to experience cesarean delivery, preeclampsia, and T2DM after delivery, and babies of GDM mothers have a higher risk of macrosomia, shoulder dystocia, birth injuries, hypoglycemia, and hyperbilirubinemia compared with those of nondiabetic mothers [4-6].

Epidemiologic studies have shown that modifiable risk factors such as prepregnancy body weight, recreational physical activity before and during pregnancy, and dietary patterns before pregnancy may be related to GDM risk [7]. GDM prevention efforts related to weight control and healthy lifestyle can potentially decrease risks of adverse outcomes for mothers and their children [8,9]. For up to 85% of women who already have a diagnosis of GDM, lifestyle changes may be sufficient to manage the disease, while oral metformin or insulin therapy might be needed for others [10]. Women with mild GDM who received dietary intervention, self-monitoring of blood glucose (BG), and insulin therapy had significantly lower risks of macrosomia (5.9% vs 14.3%, *P*<.001), shoulder dystocia, cesarean delivery (26.9% vs 33.8%, *P*=.02), and preeclampsia or gestational hypertension (8.6% vs 13.6, *P*=.01) than those who received standard care in a randomized controlled trial (RCT) of 958 women [11].

Mobile phones have portability, constant Internet connectivity, and increasing capacity to run complex apps, which makes them ideal tools in health services to collect personal information, provide personalized intervention, and potentially save time and cost as compared with standard health care [12,13]. Mobile phone apps are showing a positive impact on T1DM and T2DM self-management in the past two decades [14]. As self-management is critical for all diabetes patients, women with GDM may be highly motivated to adopt GDM self-management regimens, since they are concerned with possible complications of the disease affecting their baby [15,16]. Health-conscious pregnant women are likely to view apps and social media sites as a means to improve and monitor their pregnancy, their personal health, and their child’s development and health [17]. Although GDM apps are widely available on online app stores, few published articles have described these apps, and we know of no review of mobile phone apps for GDM being published to date.

Being diabetic during pregnancy is challenging and can create high levels of stress and anxiety. Women with GDM need to access information about the disease, make adjustments to their lifestyle habits, learn to monitor their BG, and potentially learn to administer insulin or other medication in a very short period—usually 12 to 16 weeks from diagnosis to delivery [18]. There is evidence to suggest that health literacy (defined as the degree to which individuals have the capacity to obtain, process, and understand basic health information and services needed to make appropriate health decisions [19]) is specifically associated with diabetes management. Indeed, T2DM patients with lower health literacy levels have less diabetes-related knowledge [20] and are less engaged in mobile- and Web-delivered self-care interventions [21]. Furthermore, more engagement with these interventions is correlated with better glycemic control [21]. Among women whose pregnancies were complicated with diabetes (pregestational diabetes or GDM), health literacy was associated with patient-provider communication and risks that may cause adverse pregnancy outcomes, such as not taking a folic acid supplement [22,23]. Health literacy levels also confound the delivery of care from providers to GDM patients, especially among women from a disadvantaged background. Low literacy has been shown to have a significantly negative impact on women’s understanding of GDM information and their ability to engage in a dialogue with health providers about their care. Low literacy also increases the communication challenges for diabetes educators who are working with these women [24]. Level of knowledge about GDM is significantly associated with glycemic control [25]. Literacy-appropriate and culturally appropriate educational messages should be developed and delivered to improve the health of patients and lessen the burden for their providers [24]. Mobile phone apps can be a useful educational strategy for GDM women with low health literacy due to the apps’ flexibility of providing tailored information [26]. However, to our knowledge, the health literacy feature of mobile phone apps targeting women with GDM has not been evaluated.

Our objective was to review the literature on mobile phone apps designed for women who have or are at risk for developing GDM, and to describe the development, functionality, implementation, and impact of these apps. A secondary objective was to summarize the health literacy-related features of the apps described in the identified articles.

## Methods

Research on mobile phone apps and GDM is relatively new; therefore, we conducted this scoping review as a first step to examine the availability of literature in this area. Scoping reviews are different from systematic reviews in that they answer broader research questions, and studies in various designs instead of a few predefined designs such as RCT and cohort can be relevant to the research questions; in addition, the quality of the studies is not evaluated [27]. We followed Arksey and O’Malley’s 5-stage scoping review framework [27] to (1) identify the research questions, (2) identify relevant studies, (3) select studies, (4) chart the data, and (5) collate, summarize, and report the results.

### Stage 1: Identifying the Research Questions

The research questions addressed in this review were as follows: (1) What is known from the existing literature about the availability, functionality, and effectiveness of mobile phone apps on GDM prevention and management? (2) What is the role of health literacy in these apps?

### Stage 2: Identifying Relevant Studies

We selected 7 databases in consultation with a reference librarian: PubMed, Cochrane Library, Web of Science, CINAHL Complete, Communication & Mass Media Complete, Inspec, and Google Scholar. We identified articles by conducting searches using a combination of 2 sets of keywords: (1) gestational diabetes and (2) mobile, app, digital, technology, mHealth, wearable, wireless, smartphone, cell phone, telemedicine, or telecare. The combination of the first 2 sets of keywords and a third keyword, literacy, was searched in all 7 databases to identify extra articles. To retrieve the most relevant results, titles and abstracts were searched in PubMed; title, abstract, and keywords were searched for in Cochrane Library; topics were searched for in Web of Science; and abstracts were searched in CINAHL Complete, Communication & Mass Media Complete, and Inspec. The Google Scholar search was based on title. We also conducted a backward search of references of all articles that met the inclusion criteria.

### Stage 3: Selecting the Studies

We retrieved articles for further analysis according to the following inclusion criteria: the targeted study population had to be women with GDM or women who were at risk of GDM; we considered overweight and obese women to be at risk for developing GDM. Studies had to describe a mobile phone app, and the mobile phone app had to focus on health promotion or disease prevention, or both. Exclusion criteria were studies focused solely on women with T1DM or T2DM. We excluded studies if mobile devices were used only to communicate between patients and health care providers (for data transmission, short messages, talk, or counseling). Other exclusion criteria were reviews or editorials, studies not in English, and studies for which the full text was not available.

**Figure 1:**
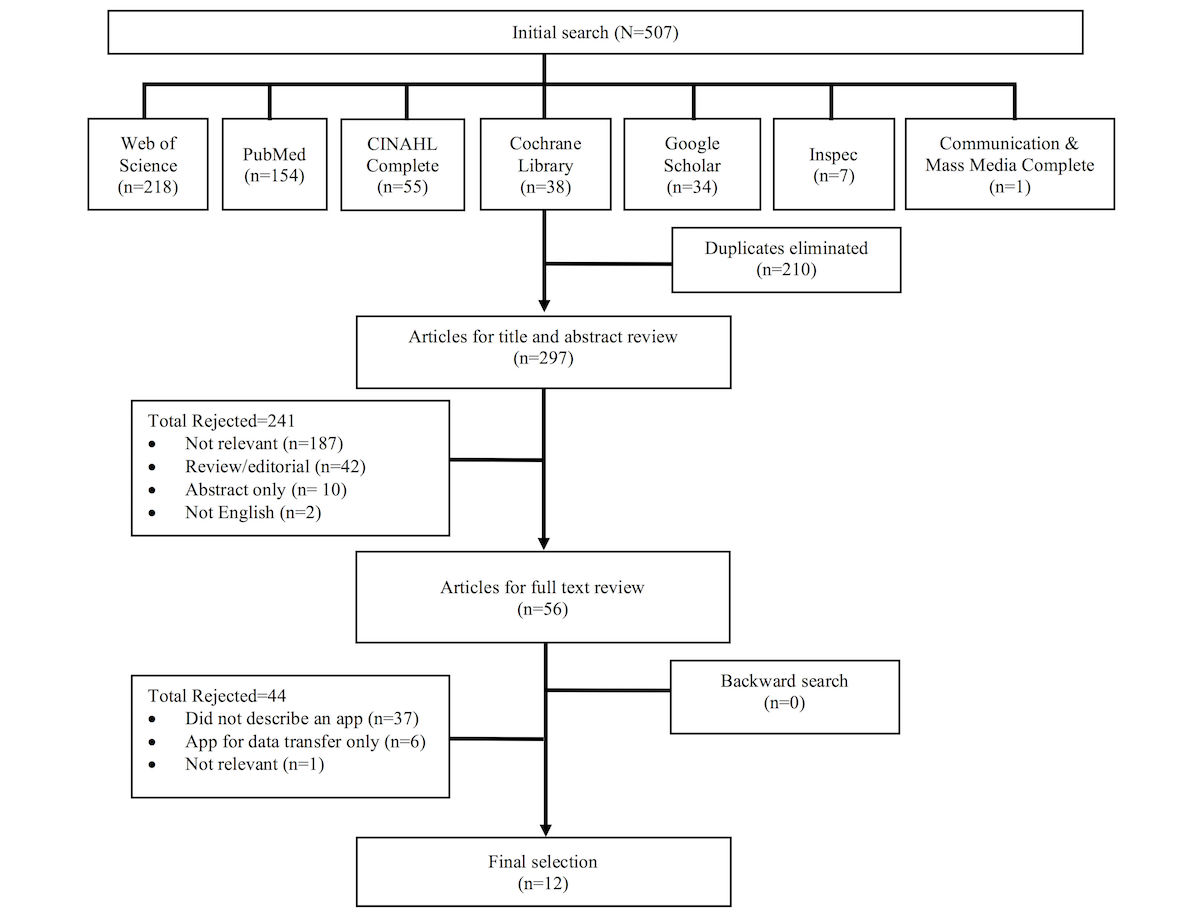
Flow diagram of the search strategy.

According to these criteria, we selected the articles by title, abstracts, and then full text. Figure 1 presents the flow diagram of the search strategy. The initial searches were carried out in July 2016 and were not limited by date. The same searches were performed again in April 2017 to identify newly published studies.

### Stage 4: Charting the Data

After a full-text review, we classified studies into 2 categories based on the following content: (1) description of the development, feasibility, or usability of the apps or systems, and (2) trial protocols. To answer the research questions, we created a data charting form in Excel for Mac Version 15.25.1 (Microsoft Corporation) with the following elements: authors, year of publication, country of the study, category of the study, features of the app, behavioral theories, personalization features of the app, health literacy-related features, study design, sample characteristics, usability, feasibility, intervention components, and outcome measures.

### Stage 5: Collating, Summarizing, and Reporting the Results

We used information from the data charting form to summarize the overall number of studies, years of publication, characteristics of the study populations, countries where studies were conducted, and the focus and purpose of the studies. We report results of the review as categories and elements identified in stage 4 to answer the research questions, make comparisons among the studies, and identify research gaps.

## Results

A total of 507 articles from the 7 databases matched the initial search criteria using a combination of the keyword gestational diabetes and app-related keywords. The addition of a third keyword, literacy, did not yield additional results. Removing duplicates resulted in 297 articles for title and abstract review. We excluded 241 articles after the abstract review for the following reasons: not relevant (n=187), review or editorial (n=42), abstract only (n=10), and not in English (n=2). We reviewed the full text of the remaining 56 articles, which resulted in the elimination of 44 articles for the following reasons: did not describe an app (n=37), the app was used only to transfer data (n=6), and not relevant (n=1). We included a total of 12 articles in the final analysis (Figure 1).

### Characteristics of the Studies

The final 12 articles were published between 2014 and 2017 and were conducted in 7 countries: Switzerland, Spain, Norway, the United Kingdom, South Korea, Ireland, and Malaysia. Among the 12 articles, 7 described the development and feasibility or usability of the app or system [28-34]; 4 proposed RCT protocols using an app or system [35-38]; and 1 described the development of an app [16]. A total of 7 distinct systems or projects containing an app were described in the 12 articles.

### Development, Usability, and Feasibility of Apps and Systems

Table 1 summarizes specific features of the apps and systems. Table 2 summarizes the results from development, usability, and feasibility studies.

Garcia-Saez et al described the development of a telemedicine system called MobiGuide [16]. This system used a decision support system (DSS) to generate personalized feedback for GDM management based on an expert-approved GDM guideline and patients’ data. MobiGuide includes a body area network, which provides real-time monitoring of biosignals such as BG by a Bluetooth-enabled glucometer, blood pressure by a blood pressure monitor, and activity level by an accelerometer within the smartphone. The MobiGuide system generates advice directly to both patients and health care providers. For the patients, advice regarding therapy, monitoring, and clinical assessment was generated based on their compliance with the therapy prescribed by the doctors to reinforce their behaviors. For the doctors, recommendations for changing diet and exercise, or insulin prescriptions, were generated based on patients’ compliance and BG control. In the feasibility test of the MobiGuide system, 20 women with GDM were initially instructed to measure their BG (4 times/day), ketonuria (once/day, manually entered), blood pressure (twice/week), and weight (once/week). One participant dropped out after 1 week. Recommendations were modified based on patients’ control and compliance. For example, good glycemic control could switch the BG measurement recommendation from 4 time per day to twice per week with 4 measures per day. Patients had high compliance (proportion of performed to recommended numbers of measurements) for BG (0.87±0.11), ketonuria (0.98±0.03), and blood pressure (0.82±0.24). When compared with the data of a historical cohort of 247 GDM patients, MobiGuide users had significantly better compliance to follow at least 4 BG measurements on indicated days (1.01±0.10 vs 0.87±0.28; *P*=.03) and better blood pressure control (98.6/64.7 vs 119.3/72.8 mmHg; *P*<.001). All 19 patients who completed the study received the message “High BG (2 abnormal per week). Did you eat more than you should?” The compliance rate (number who responded to the questions) was 0.31. Other recommendations related to ketonuria were generated to fewer patents (1-4 patients) with 0 to 1 compliance. Most of the users (12/17, 71%) thought the system improved their confidence in GDM management, 15/17 (88%) thought it did not complicate their lives, 12/17 (71%) liked the system’s ability to adapt to their daily life and context change, and 16/17 (94%) would recommend it to other patients. All 6 clinicians thought the system helped them to identify treatment priorities, 5/6 (83%) agreed the system increased patient safety, and 4/6 (67%) thought the system made it easier to manage patients [34].

As part of the expert personal health system (PHS) developed by Bromuri et al, an Android smartphone was given to GDM women to input their BG measures once they read it from the glucometer that they were provided [28]. Autonomous software entities, or agents, were programmed based on the American Academy of Family Physicians’ monitoring rules [39] to serve as the “experts.” The PHS was able to monitor BG readings and generate text-based alerts of hypoglycemic and hyperglycemic events to the caretaker (nurses, dietitians, doctors) on a Web interface. For example, if 2 BG values were less than 3 mmol/L within 1 hour, a hypoglycemia warning would be generated. With the automated alerts, caretakers were able to initiate an in-person or phone-based consultation with the patient based on the BG measures that triggered the alerts. An RCT was conducted with 24 GDM women to compare effects between a control group (standard care, n=12) and an intervention group (n=12) using the PHS in addition to standard care [28]. All women were asked to record their BG values 6 times a day for 2 to 4 months: fasting, postprandial breakfast, preprandial lunch, postprandial lunch, preprandial dinner, and postprandial dinner. The intervention group recorded significantly more BG measurements than the control group (235±86 vs 135±80; *P*<.001). Women in the intervention group had overall better BG control (5.4 vs 5.7 mmol/L or 98 vs 102.4 mg/dL; *P*<.001) than the control group. Among the 6 daily BG measurements, 4 were significantly better controlled in the intervention group (all 4 *P*<.001). In the intervention group, all patients rated the smartphone app easy to use and were satisfied with the care provided by the system. Caregivers in this study considered the system appropriate for GDM management. Although the time needed for patients’ consultation remained the same, the caregivers thought the PHS was more time efficient because they were able to focus on the hyperglycemic or hypoglycemic events based on the alerts instead of going through patients’ BG records manually. In addition, consultations could be initiated within 1 to 3 days with the PHS instead of 1 to 3 weeks with the standard care after the hyperglycemic or hypoglycemic events. The PHS system also allowed for the possibility of daily consultation on patients’ BG readings.

**Table 1:** Characteristics of the 7 apps and systems.

Author, year, reference	Country	App and technology characteristics	Theory and theoretical constructs	Personalization	Health literacy-related features
Garcia-Saez, 2014 [16]	Spain	MobiGuide (app) Patients’ data automatically collected by body area network or manually entered in app; decision support system generates feedback to patients and clinicians based on clinical guidelines.	Reminders and advice generated to reinforce behaviors.	Patients’ compliance, BG^a^ control, personal information, and preferred time of receiving reminders used to generate personalized reminders.	N/A^b^
Bromuri, 2016 [28]	Switzerland	PHS^c^ (app and Web) Patient’s side: Android app collects BG values, medication data, and symptoms. Caregiver’s side: a Web app, existing medical knowledge designed to provide alerts about the glycemic values to caregivers.	N/A	Alerts based on patients’ BG control.	BG data visualization.
Garnweidner-Holme, 2015 [29]	Norway	Pregnant *+* (app) Auto transfers BG levels; gives immediate feedback on BG levels; provides information about healthy eating and PA^d^; prints BG records at their clinics; provides general information about GDM^e^.	Health belief model used to develop content.	Culturally tailored dietary recommendations; information tailored to preference and prepregnancy PA level.	Content checked against Suitability Assessment of Materials and Kreuter’s message checklist to improve text and layout. A diabetes lexicon was used to explain medical jargon. BG data visualization.
Jo, 2016 [32]	South Korea	App generates common recommendations applicable to all GDM patients and tailored recommendations based on algorithms linking patients’ data and clinical guidelines.	N/A	Tailored recommendations based on BG, diet, PA, ketone, and weight.	N/A
Mackillop, 2014 [33]	United Kingdom	GDm-Health (system) Automatically uploads BGs from glucometer to app through Bluetooth and then to server; health care professionals have remote access; 2-way communication between women and health care professionals.	N/A	Alerts generated by the system to health care providers based on frequency and reading of BG.	BG data visualization.
Kennelly, 2016 [36]	Ireland	Pears (app) Provides list of daily PA and behavioral tips, and a database of low glycemic index recipes.	Control theory: SMART^f^ goals; social cognitive theory: barriers to change	Dietary advice and PA goals set at in-person education session with nutritionist or dietitian and obstetrician.	N/A
Skau, 2016 [38]	Malaysia	Jom Mama eHealth platform (app and Web) App incorporates personal goal setting, progress tracking, and general information on healthy lifestyles. A Web-based back-end interface can be accessed by CHPs^g^.	Goal setting with CHPs and in the app, motivational interviewing techniques adopted by CHPs.	Personalized goal setting and follow-up with CHPs. The app provides interactive options allowing users to select lifestyle challenges.	Change in health literacy is a secondary end point.

^a^BG: blood glucose.

^b^N/A: not applicable.

^c^PHS: personal health system.

^d^PA: physical activity.

^e^GDM: gestational diabetes mellitus.

^f^SMART: specific, measurable, achievable, relevant, and time specific.

^g^CHP: community health promoter.

**Table 2:** Summary of usability and feasibility studies and RCT^a^ protocols.

Author, year, reference	Country	App or system name	Focus and study design	Target audience and sample	Key results and outcome variables
Peleg, 2017 [34]	Spain	MobiGuide	Feasibility Quasi-experimental	Intervention: GDM^b^ patients (n=20) Control: historical cohort GDM patients (n=247) Duration: <34th gestational week to delivery (5-11 weeks)	Intervention vs control: BG^c^ measurement compliance^d^ (1.01±0.10 vs 0.87±0.28; *P*=.03), BP control (98.6/64.7 vs 119.3/72.8 mmHg; *P*<.001). Patient compliance^e^: BG measures (0.87±0.11), ketonuria (0.98±0.03), BP (0.82±0.24), responded to message “High BG (2 abnormal per week), did you eat more than you should?” (0.31). Patient satisfaction (rated positive): system increased confidence (12/17), liked system’s adaptability to daily life (12/17), system did not complicate life (15/17); would recommend to others (16/17). Clinician satisfaction (rated positive): system helped identify priorities (6/6), increased patient safety (5/6), easier to manage patients (4/6).
Bromuri, 2016 [28]	Switzerland	PHS^f^	Development, usability, feasibility RCT	Intervention (telemedicine): GDM patients (n=12) Control (standard protocol): GDM patients (n=12) Duration: 24th-32nd gestational week to delivery (2-4 months)	Intervention vs control: number of BG measures (2749 vs 1616; *P*<.001); BG control (5.4 vs 5.7 mmol/L or 98 vs 102.4 mg/dL; *P*<.001). Intervention group satisfaction: 12/12 satisfied with the care by PHS and perceived the system easy to use. Caregiver satisfaction: perceived the system as appropriate, reduced reaction time, provided possibility of daily consultation, and saved time through automated alerts.
Garnweidner-Holme, 2015 [29]	Norway	Pregnant+	Development, usability	Women with GDM (N=22) Duration: 1-time use of the app	Perceived ease to register and control BG levels. Participants had success performing given tasks: finding information on healthy eating (10/11), physical activities (10/11), GDM (10/11), finding where to register BG levels (11/11), entering appointments for medical consultations (9/11), and finding how to register body weight (5/11).
Borgen, 2017 [35]	Norway	Pregnant+	RCT protocol (ongoing)	Women with a 2-hour OGTT^g^ ≥9 mmol/L (N=230) Intervention: app + standard care Control: standard care Duration: <33rd gestational week to 3 months postpartum	BG level measured at 2-hour OGTT 3 months postpartum. Change in health behavior and knowledge about GDM, quality of life, birth weight, mode of delivery, and complications for mother and child.
Jo, 2016 [32]	South Korea		Development, usability, feasibility	Usability: GDM patients (n=5) User acceptance test: GDM patients (n=60) Duration: 1 week	Average usability score: 69.5 out of 100. User acceptance score with behavioral intention to use 5.5, intrinsic motivation score 4.3, perceived ease of use score 4.6, and perceived usefulness score 5.0, out of 7 for all measures.
Mackillop, 2014 [33]	United Kingdom	GDm-Health	Development	Beta testing phase: GDM patients (n=7) Service development phase: GDM patients (n=50) Duration: diagnosis to delivery	Women used the system for 13.1 weeks on average. 46/54 women submitted the minimum of 18 BG readings per week. 19,410/19,686 (98.6%) of BG readings were manually tagged with additional information (time of measurement and comments) by patients.
Hirst, 2015 [30]	United Kingdom	GDm-Health	Usability	See row above	Satisfaction: women were satisfied with the care (45/49), and agreed the equipment was convenient (47/49), reliable (43/49), and fit into their lifestyle (42/49).
Hirst, 2016 [31]	United Kingdom	GDm-Health	Feasibility	See 2 rows above	12/41 (29%) women delivered LGA^h^ babies. Mother’s BG (LGA vs non-LGA babies): mean BG (6.3 vs 5.6 mmol/L; *P*=.004), fasting BG (5.8 vs 5.1 mmol/L; *P*=.004), and 2-hour postprandial BG (6.9 vs 6.0 mmol/L; *P*=.001). Odds of delivering an LGA baby increased with every 1-SD increase (0.7 mmol/l) in mean BG (OR^i^ 5.5, 95% CI 1.4-21.2) and mean postprandial BG (OR 6.1, 95% CI 1.6-23.4).
Mackillop, 2016 [37]	United Kingdom	GDm-Health	RCT protocol (ongoing)	N=200 pregnant women with abnormal glucose tolerance Intervention: use GDm-Health system (app), attend the clinic every 4-8 weeks Control: standard care, self-record BG diary at home, attend the clinic every 2-4 weeks Duration: 14-34 weeks to delivery	Efficacy of GDm-Health; BG control and management intensity; maternal and fetal outcomes.
Kennelly, 2016 [36]	Ireland	Pears	RCT protocol (ongoing)	N=506 pregnant women, 10-15 weeks’ gestation, body mass index 25-39.9 kg/m^2^ Intervention: targeted low GI^j^, nutritional advice, and a daily exercise prescription (in-person education session) with a smartphone app as support, and biweekly follow-up emails Control: standard obstetric care Duration: 2nd to 3rd trimester	Incidence of GDM at 29 weeks. Gestational weight gain, maternal physical activity levels in the 3rd trimester, and GI and glycemic loading of maternal diet in the 3rd trimester.
Skau, 2016 [38]	Malaysia	Jom Mama	RCT protocol (ongoing)	N=660 newly registered married or engaged couples. Female not pregnant, diabetes-free at baseline Intervention: contact with community health promoter: 3 face-to-face meetings, 3 phone calls, communication through WhatsApp group chat, and use of the eHealth platform Control: standard care Duration: 8 months	Change in abdominal fat content. Change in body mass index, waist-to-height ratio, waist-to-hip ratio, weight, hemoglobin A_1c_, fasting lipid profile, blood pressure, health literacy, dietary intake, physical activity and sedentary behavior, and stress level. Incidence of GDM.

^a^RCT: randomized controlled trial.

^b^GDM: gestational diabetes mellitus.

^c^BG: blood glucose.

^d^Number of days measured ≥4 BGs/number of days prescribed to measure BG.

^e^Proportion of performed/recommended measurements.

^f^PHS: personal health system.

^g^OGTT: oral glucose tolerance test.

^h^LGA: large for gestational age.

^i^OR: odds ratio.

^j^GI: glycemic index.

Pregnant+ is an app developed to monitor GDM women’s BG level by Bluetooth transmission or manual input [29]. It generates immediate feedback, provides information on healthy diets based on the cultural background of the user (eg, using food items preferred in users’ cultures), provides physical activity information based on level of activity, and provides general information about GDM. The content of the app was designed to emphasize patients’ perceived severity of their disease, emphasize perceived benefits to treatment and management, and provide cues to action based on the health belief model. Although BG records cannot be transferred automatically to the health care providers due to medical data security, users can print their BG records at the clinics [29]. In a user involvement study for the Pregnant+ app, most participants were able to perform tasks related to the 4 major functions of the app; namely, finding (1) where to register BG levels (11/11, 100%), (2) information about healthy eating (10/11, 91%), (3) information about physical activities (10/11, 91%), and (4) general information about GDM (10/11, 91%) [29]. Fewer participants were able to find other functions, such as entering appointments for medical consultations (9/11, 82%) and finding how to register body weight (5/11, 45%). Users of this app believed it would make it easier for them to register and control their BG level than with standard care. They also reported favorable reviews for the features that provided real-time feedback and information about GDM, diet, and physical activity.

Jo and Park developed an app for Korean women with GDM [32]. This app generates recommendations about the risk factors of GDM, importance of GDM management, and management of BG, diet, physical activity, and body weight to patients based on their initial assessment and lifestyle data, including caloric intake and physical activity level. Algorithms using patients’ data and clinical guidelines [40-42] were developed to generate individually tailored recommendations. A total of 5 GDM patients participated in the usability test of this app. The average usability score was 69.5 out of 100 as measured by a Korean version of the System Usability Scale [32]. User acceptance was measured using Wilson and Lankton’s model of patients’ acceptance of provider-delivered eHealth [43]. The user acceptance score with behavioral intention to use was 5.5, intrinsic motivation score was 4.3, perceived ease of use score was 4.6, and perceived usefulness score was 5.0, out of 7 for all measures.

The GDm-Health system is a real-time BG monitoring management system for women with GDM that consists of a smartphone app and a website [33]. This smartphone app allows women to automatically synchronize their BG levels from their glucose meter through Bluetooth and provides immediate feedback based on the BG readings. BG levels are sent to a central server where health care professionals can access the data on a website. Another function of this system is to allow 2-way communication where health care professionals give advice or change medication and users can request a callback from the team to address their concerns. A total of 7 women were involved in the beta test phase of the GDm-Health system, and 50 women with GDM tested the system until delivery. On average, the women used the system for 13.1 weeks, 46 of 54 (85%) submitted the minimal requirement of 18 BG readings per week, and 19,410 of 19,686 (98.6%) readings were manually tagged with additional information indicating when it was measured (pre- or postprandial) [33]. The Oxford Maternity Diabetes Treatment Satisfaction Questionnaire was developed and used to assess the acceptability of the system [30]. Overall, 45/49 (92%) women were satisfied with the care delivered by the system, and 46/49 (94%) agreed they had a good relationship with their care team. Most agreed that the equipment was convenient (47/49, 96%), reliable (43/49, 88%), and fit into their lifestyle (42/49, 86%). Birth outcome data were available for 41 women, of whom 12 (29%) delivered large for gestational age (LGA) babies. Mothers of LGA versus non-LGA babies had significantly higher mean (6.3 vs 5.6 mmol/L; *P*=.004), fasting (5.8 vs 5.1 mmol/L; *P*=.004), and 2-hour postprandial BG readings (6.9 vs 6.0 mmol/L; *P*=.001). A 1-SD increase (0.7 mmol/L) in mean BG increased the odds of delivering an LGA baby by fivefold (odds ratio 5.5, 95% CI 1.4-21.2) [31].

### Randomized Controlled Trial Protocols

A total of 4 ongoing RCTs are using a mobile phone app or using an app as part of the intervention component to prevent or manage GDM [35-38]. Table 1 summarizes characteristics of the apps and Table 2 summarizes characteristics of the RCT protocols.

Mackillop et al are testing the efficacy of using the GDm-Health system compared with standard clinic care [37]. A total of 200 women with abnormal glucose tolerance between 14 and 34 weeks of gestation have been randomly assigned to 1 of 2 groups: GDm-Health system and clinic visit every 4 to 8 weeks; or normal clinic care (visit the clinic every 2 to 4 weeks). The primary outcome is BG control, as determined by mean BG readings from recruitment until delivery compared between the intervention and the control group. The secondary outcomes are compliance with the allocated BG monitoring regimen, maternal and neonatal outcomes, glycemic control using hemoglobin A_1c_ and other BG metrics, and patient attitudes toward care.

Borgen and colleagues are evaluating the efficacy of the Pregnant *+* app [35]. A total of 230 pregnant women with GDM who own a smartphone, understand Norwegian, Urdu, or Somali, and are before 33 weeks of gestation were recruited. Women will randomly receive either the Pregnancy+ app and standard care or standard care until 3 months postpartum. The primary outcome is glucose tolerance after the intervention, measured by 2-hour oral glucose tolerance test. Secondary outcomes are birth weight, mode of delivery and complications for mother and child, change in diet and physical activity from baseline to 36 weeks of gestation (measured by a modification of the Fit for Delivery questionnaire and the Pregnancy Physical Activity Questionnaire), and quality of life (measured by a short version of the Edinburgh Postnatal Depression Scale and by health-related quality of life during pregnancy and postpartum) [35].

Kennelly et al are conducting a pregnancy, exercise, and nutrition research study with smartphone app support (Pears) targeting low glycemic index dietary and physical activity promotion among overweight and obese pregnant women [36]. The Pears healthy lifestyle package includes a 75-minute in-person education session, biweekly emails, 2 follow-up appointments, and an app. The app provides behavior, dietary, and physical activity tips, physical activity benefits, and a database of low glycemic index recipes. Control theory and social cognitive theory were applied to set up patients’ personal SMART (specific, measurable, achievable, relevant, and time-specific) goals, and to overcome personal and environmental barriers to change. The authors are randomly assigning 506 overweight or obese women between 10 and 15 weeks’ gestation to the intervention or the control arm to assess the impact of the Pears healthy lifestyle package [36]. The primary outcome is the incidence of GDM at the 29th week. Secondary outcomes will be gestational weight gain, maternal physical activity levels, and glycemic index and glycemic load of the mothers’ diet in the third trimester.

Skau et al developed a behavior change intervention, Jom Mama, targeting young Malaysian couples to promote women’s health prior to pregnancy [38]. This project includes 3 in-person and 3 phone communications with community health promoters, and an eHealth platform. The eHealth platform includes an app for the couples and a Web-based interface for the community health promoters. The couples can set personal goals, track their progress, and access general information on healthy lifestyles from the app. Women and spouses can select different challenges for obtaining a healthy diet (eg, avoid soft drinks), increasing their physical activity, or decreasing their sedentary behavior during the intervention period (eg, cycle for 30 minutes). Community health promoters follow the progress of the couples and interact with them during the in-person and phone communications using the information from the Web-based interface. Skau and colleagues are testing the efficacy of the Jom Mama project on preconception health promotion [38]. They are recruiting a total of 660 nulliparous women between 20 and 39 years of age who own a smartphone and are free of diabetes to randomly assign to an intervention or control group. The planned follow-up duration will be 8 months. The primary outcome is change in waist circumference. Secondary outcomes will be changes in other anthropometric (eg, body mass index, waist-to-hip ratio), biochemical measures (eg, hemoglobin A_1c_, lipid profile), health literacy, dietary intake, physical activity, and stress level. They will also measure the incidence of GDM proposed as an outcome in women who completed the intervention and become pregnant after the trial.

### Health Literacy-Related Features

Health literacy was taken into account in 2 of the 7 final apps and systems. The Pregnant+ app was the only system that incorporated user literacy level in the development phase [29]. The researchers checked the app against Kreuter’s message checklist, which includes checking the content, writing, literacy, and elements of visual communication [44] and administering the Suitability Assessment of Materials instrument [45] to make sure the app was appropriate for their targeted audience. In addition, after the second stage of the user involvement study with 11 GDM patients of varying literacy levels, a diabetes wordlist was added to the app to explain medical jargon [29]. The Jom Mama intervention was designed to measure change in the level of health literacy using the European Health Literacy Survey Questionnaire (a 47-item scale covering 3 domains: health care, disease prevention, and health promotion) as one of the outcomes of the intervention [38]. PHS, Pregnant+, and GDm-Health all used a data visualization strategy to present normal and abnormal BG data in figures [28,29,33]. Although Jo and Park involved users in the development of the app, they did not mention whether the app met the health literacy level of their targeted users [32]. Mackillop et al assumed that their target users for the GDm-Health system being recruited from a large single-center tertiary referral unit in southern England would have high rates of literacy and low levels of social deprivation [37].

## Discussion

### Principal Results

There is a limited body of published data on the use of mobile phone apps for GDM. In this review, 12 articles focused on the development, usability, feasibility, and trial protocols of mobile phone apps or interventions including an app. DSSs were used to connect patients’ data to tailored feedback for both patients and health care providers using clinical guidelines. Health literacy was considered a feature of 1 app during the development phase [29] and was measured as an outcome by another app [38].

### Comparison With Prior Work

**Figure 2:**
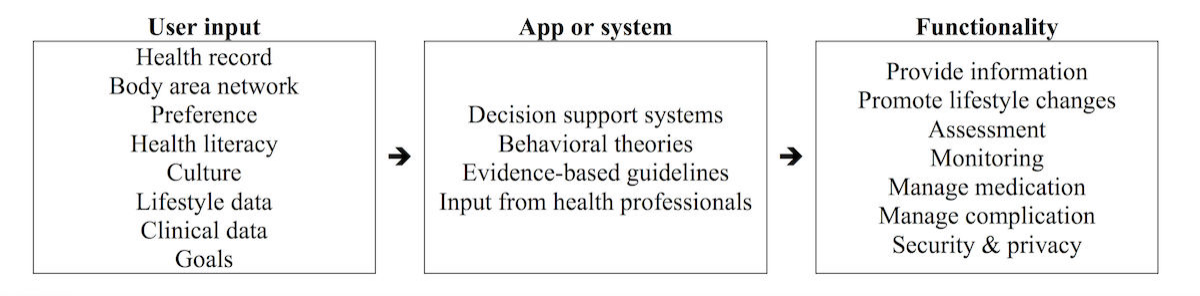
Framework of automated app or system.

Figure 2 presents the common framework combining all the characteristics of the app or system with automated features. The findings suggest that DSSs have the capacity to generate real-time personalized feedback based on users’ input and existing clinical guidelines. From the studies identified, a variety of data from the users have been collected, recorded, and saved in the app or system, or used to develop the app or system. These data include health records, biosignals collected by body area network, user preferences, culture, lifestyle data, clinical data, and personal health goals. However, not all of the information was used in the DSSs to generate output to the patients or health care providers. The information that was used most frequently was BG levels collected by body area network or entered by the users. The MobiGuide system had the highest level of personalization among all the apps and systems we identified [16]. This system feeds the DSS with historical clinical data, the personal health record, body area network sensor data, and manually entered data to generate personalized feedback. However, deciding on the right amount of advice to reinforce users’ behaviors without overwhelming them can be challenging. The primary functions of the apps and systems included providing information, promoting lifestyle change, assessing and monitoring user status, and managing medication and complication with approval from providers. Also, protecting the security and privacy of patients’ data is a common feature of the apps and systems. Similar to the findings of this review, in a review of commercial apps for diabetes self-management, El Gayar found that 12 of 71 (17%) had decision support capabilities and all of them were related to insulin dosage suggestions as opposed to lifestyle changes [14]. Internet-based interventions that promoted lifestyle modifications for diabetes management, were based on theory, included interactive components and personalized feedback, and provided peer support were most successful [46]. In our review, only 2 apps incorporated behavior change theories [16,29], and 2 RCTs used theories in their proposed interventions, but not specifically in their app or system [36].

Even if the apps provide high-quality, evidence-based content, the value is limited if the information does not adequately match and address the usability, accessibility, readability, and health literacy needs of target audiences [47]. Although users were frequently involved in usability and feasibility studies to inform the development and finalization of the app or system, their literacy level was discussed in only 2 studies. Caburnay et al analyzed the health literacy-related features of more than 100 diabetes apps with a specific focus on using plain language, displaying content clearly, organizing and simplifying the user interface, and engaging users [48]. A total of 84% of the apps employed at least one of the plain language strategies, such as using common everyday words; avoiding undefined technical or medical terms; and using active voice, action words, and present tense [48]. Involving users in the development phase and evaluating users’ attitudes, knowledge, beliefs, and behavior related to health information are helpful strategies to improve usability of an app [49].

Overall, the users involved in the usability and feasibility studies found it easy to navigate the apps and systems, and were satisfied with the technology. Apps and systems have the potential to improve compliance with BG monitoring and treatment prescriptions, and improve communication between users and health care providers. El-Gayar et al categorized the technology for diabetes self-management into the Internet, cellular phones, telemedicine, and decision support techniques [50]. However, our review found that DSSs can be embedded in mobile phone apps to generate real-time feedback.

Only 1 feasibility study [28] with a sample of 24 women showed that patients who received PHS care had better BG control than did patients who received standard care. Better blood pressure control was reported in another feasibility study; however, no difference in BG control was observed [34]. Large RCTs are needed to confirm the system’s impact on BG control and other clinical outcomes. In this review, we identified 3 RCT protocols: only 1 protocol, by Mackillop et al [37], evaluated the efficacy of the GDm-Health system, and the other 2 protocols evaluated complex lifestyle programs with an app as part of the intervention [36,38].

### Limitations

We applied no evaluation criteria to the articles due to the nature of this scoping review. This review only searched abstract, title, and topics in most databases, which may not yield a complete pool of relevant articles. Articles published in languages other than English were not included. However, this is, to our knowledge, the first known review of mobile phone apps on GDM to provide an overview of the literature.

### Conclusions

This scoping review describes the literature on mobile phone apps for GDM prevention and management. We identified and described 12 articles that discussed the design and development, usability, feasibility, and RCT protocols of GDM-related apps. Findings from this scoping review suggest that mobile phone apps have the potential to prevent GDM and improve GDM management. Future research should focus on large RCTs of the impact of these apps. In addition, health literacy levels of the potential audience should be taken into consideration when developing and evaluating the usability of apps for this audience.

## Abbreviations

BG: blood glucose
DSS: decision support system
GDM: Gestational diabetes mellitus
LGA: large for gestational age
PHS: personal health system
RCT: randomized controlled trial
T1DM: type 1 diabetes mellitus
T2DM: type 2 diabetes mellitus
